# It Takes a Team—Enhancing Student-Athlete Health and Well-Being through an Interprofessional Approach

**DOI:** 10.3390/sports12080209

**Published:** 2024-07-30

**Authors:** Rebecca Steins, Anthony P. Breitbach, Michael Ross, Erica Ciarlo, Elena Melillo, Olivia Brant

**Affiliations:** 1 Department of Psychology, College of Arts and Sciences, Saint Louis University, 3700 Lindell Blvd, St. Louis, MO 63108, USA; michael.ross@health.slu.edu; 2 Director of Interprofessional Education, Saint Louis University, 1312 Carr Lane Avenue, Suite 110, St. Louis, MO 63104, USA; anthony.p.breitbach@slu.edu; 3 University Athletics Department, Saint Louis University, 1 S Compton Ave, St. Louis, MO 63103, USAelena.melillo@slu.edu (E.M.); olivia.brant@slu.edu (O.B.)

**Keywords:** university athlete, competitive sport, interprofessional, health, mind, psychology

## Abstract

Student-athlete well-being is a key objective for individuals working with or for university athletic departments. This paper will describe how a university athletic department used a team approach to enhancing student-athlete health and well-being. The Interprofessional Education Collaborative (IPEC) Core Competencies of (1) Values and Ethics; (2) Roles and Responsibilities; (3) Communication; and (4) Teams and Teamwork provide a guiding framework for interprofessional collaboration. (IPC; Interprofessional Education Collaborative, 2023). However, significant barriers exist in implementing IPC in university athletic departments and little research exists on how to overcome these barriers in university athletic departments to enhance student-athlete wellness. To address this gap, this paper will first provide a review of the literature on athlete well-being, followed by an applied section that describes the experience of an interprofessional wellness team (IWT) consisting of a clinical sports psychology doctoral student, a licensed mental health professional, an athletic trainer, and a sports dietitian. A case vignette is used to demonstrate how IPEC core competencies are operationalized by the team to address athlete health and well-being through IPC. Recommendations on the further implementation of IPC centered around student-athlete well-being will be provided.

## 1. Introduction

Recent years have seen a greater urgency to support the well-being of university student-athletes. Both research and social commentary over the last decade has focused on the growing mental health struggles of this population, from increases in student-athlete suicide to growing advocacy for mental health care in university and professional settings. These concerns have always existed for student-athletes struggling under the pressure of existing in the sphere of high-level athletics; however, recent shifts in cultural norms around mental health and the 2020 pandemic have truly brought athlete well-being to the forefront. The pandemic had a significant impact on athletes’ well-being when athletes were pulled from their sport and isolated from their support systems. Subsequently, it is not surprising that we have seen a significant rise in mental health issues such as gambling, sleep disturbances, substance abuse, disordered eating, mood disorders (e.g., anxiety, depression), and suicidality [1]. Paralleling the increase in mental health struggles is the rise in awareness and education around athlete mental health. This growing understanding of what it means to be an athlete has resulted in decreased stigma around mental health symptoms and help-seeking behaviors in this population. While this stigma is still significantly higher in sports than in other populations due to cultural norms of not showing weakness and playing through pain in sport, we have seen a significant increase in advocacy for access to care and organizations dedicated to providing support for student-athletes [2,3]. Through this, a call for proactive, not just reactive, care for athletes has been heard around the globe, and particularly in the United States by the National Collegiate Athletic Association (NCAA), through the development and release of the NCAA Mental Health Best practices [4]. Athlete well-being has quickly become the focus of collegiate athletic departments across the country, with both clinical and research focuses shifting in the direction of promoting the overall health and well-being of the athlete.

In line with this shift, the following paper will first address the research that currently exists on athlete well-being, the factors that influence it, and how interprofessional collaboration can be utilized to promote athlete wellness. The second portion of the paper will describe an NCAA Division I interprofessional care team, the barriers they experienced, and how they overcame these barriers within an IPC competency framework. A case vignette will be used to provide practical suggestions for implementing interprofessional care in a university athletic department to promote student-athlete well-being.

## 2. Student-Athlete Well-Being

Due to the seemingly limitless factors that can and do play a role in one’s well-being, the concept of well-being has been defined in multiple ways [5,6]. Navarro and colleagues succinctly describe student-athlete well-being as “[it] goes beyond a successful athletic performance. Student-athlete well-being can be described as pertaining to an athlete’s physical health, mental health, stigma, athletic performance, and self-care” [3] (p. 86). For student-athletes, this also encompasses interpersonal, academic, and athletic growth.

Athlete well-being is influenced by interpersonal, intrapersonal, and societal factors. At the relational level, student-athletes are influenced daily by coaches, teammates, support staff, administrators, professors, family members, peers, and friends. Research carried out on the influence of relational pressures has found that athletes who strive to please or meet the expectations of significant others in their life, such as family, friends, and coaches, are at increased risk for lower self-esteem and sustaining an injury [7]. Cho and colleagues reported that higher perceived teammate support was significantly related to higher subjective well-being [8]. Similarly, Lemelin and colleagues describe the importance of coaches and parents in promoting autonomy in their athletes to improve well-being [9]. The trust and support student-athletes perceive from sports and academic administrators also have significant influence on their well-being. Additionally, administrators tend to make final decisions on resource allocation that can directly impact their athletes [2,3]. Finally, support staff such as athletic trainers, sports medicine physicians, sports performance, and sports mental health professionals play a major role in promoting physical and mental well-being for the athletes they come into contact with [3].

Intrapersonal factors also have a significant impact on perceived athlete well-being. Sauve and colleagues identify a number of individual factors that elite athletes ascribe as influencing their well-being, which we will expand to the context of university student-athletes [10]. First, athletes whose mindset focuses purely on results and winning tend to see direct relationships between success, self-worth, and consequently, well-being. An athlete who focuses on extrinsic factors may perceive loss as a failure of the self, which negatively affects well-being—this may be in the classroom, in relationships, or in sports [10]. And second, athletes who overidentify with norms related to the sports ethic—such as taking risks, sacrificing, and playing through pain—tend to normalize issues that negatively influence well-being. These include normalizing pain, physical weakness/overtraining, excessive fatigue, and frustration/aggression towards self or others [11]. Moreover, overidentification with these norms places athletes at a significantly increased risk for injury; injury is continuously reported as one of the most detrimental experiences an athlete experiences with regard to their sense of self and well-being [12,13].

An additional influence on athletes’ well-being is their personal identity characteristics. In a sport world that is growing in diverse identities and cultural backgrounds, student-athletes’ well-being is being impacted at a multifaceted level that must be considered when addressing their mental and physical health. Student-athletes who have little autonomy or freedom built into their daily schedules are at increased risk of feeling isolated from the community outside of their immediate sport context [10]. In the case of athletes of minoritized identities, such as BIPOC, queer, or gender diverse athletes, they may feel isolated in their sport and feel unable to connect to resources or community outside of their sport due to the time demands and lack of autonomy student-athletes experience. Lack of connection and perceived social isolation are detrimental to the well-being of athletes, but especially minoritized athletes in predominantly white, cisgender, heterosexual spaces in sports.

Women-identifying, transgender, and nonbinary athletes face unique pressures in the face of a male-dominated sport context that values highly masculine traits [14,15]. Additionally, because men have historically been the gatekeepers to sport, most resources developed for athletes have been developed with cisgender men, including mental health interventions used to increase athlete well-being. DeFreese and colleagues reported that while sports participation is correlated with improved well-being, quality of life, and psychological outcomes for male athletes, this finding did not translate for female athletes [16]. They call for interventions developed specifically for female athletes to improve these outcomes. Sexual orientation and gender identity may also have different impacts on well-being. While more athletes are expressing their sexuality, a stigma around queerness still prevails, especially in male-dominated sports [17]. Moreover, the rhetoric around transgender athletes remains significantly impairing for trans athletes, resulting in negative psychological outcomes and decreased sports participation [15,18]. Black and Indigenous people of color (BIPOC) individuals also face unique challenges as athletes that impact well-being. Researchers describe how racial disparities in sports influence moral development and well-being, reporting that Black athletes who participate in NCAA revenue sports (e.g., football and basketball) report increased perceived pressure that negatively impacts their daily functioning [7]. Moreover, socioeconomic status, both during childhood and into college-aged years, can create disparities in resource allocation, access to training facilities, equipment, or the ability to work with sports professionals such as a sports psychologists [19].

And lastly, in addition to personal identity factors, broader cultural values in sports can decrease the well-being of student-athletes. The sports ethic continues to create stigma related to mental health concerns in athletes, casting a light on these issues as a form of weakness, rather than a common experience of most college-aged students and peers [14]. This stigma decreases help-seeking behaviors, leaving athletes to manage these feelings on their own in spaces that feel unsupportive and at times exacerbate the symptoms. Moreover, student-athletes report that there continues to be an increase in pressure to perform across multiple facets. There is the pressure to compete and win for their own self-worth, support of coaches, and university revenue, pressure to perform academically, pressure to make NIL (name, image, and likeness) financial deals, and pressure to balance all of these different factors all while being in the early stages of their adult life [7,10]. In short, examining all of the factors presented here, there is little doubt that being a student-athlete in this current state has the potential to cause a deterioration of their mental health and well-being. As a result, student-athlete well-being is being prioritized in university athletic spaces now more than ever. One such way athletic departments are accomplishing this is by developing interprofessional care teams dedicated to athletes’ well-being.

## 3. Building Better Care

The NCAA Sport Science Institute released the second iteration of their Mental Health Best Practices in early 2024, providing NCAA athletic departments with specific targets to reach in order to best support the well-being of their student-athletes [4]. These best practices include such things as mental health protocols, referral procedures, improved screening for mental health problems, and increased access to licensed professionals. As part of their recommendations, they highlight the need for interprofessional care teams to help prevent, treat, and support mental health concerns in student-athletes [4,10]. A diverse team of professionals is needed for treating athletes’ post mental health crisis such as a licensed mental health professional, psychiatrist, sports physician, etc. However, these resources are currently stretched thin in athletic departments across the country as care demands continue to increase due to the multitude of factors described in the preceding section [2]. Therefore, the focus must shift to preventative care—how we can promote well-being to prevent athletes from reaching a crisis point where significant mental health resources are needed. A stepwise care model is being successfully utilized in medical settings [20] and in mental health care systems [21] to engage in preventative work at both the individual and community levels with success at reducing negative medical and mental health outcomes. Part of this approach includes incorporating professionals from varying backgrounds to address the variety of factors that can impact well-being before they escalate. In athletic settings, the care team can consist of persons from various professional backgrounds, including athletic trainers, sports psychologists, mental health therapists, sports physicians, strength and conditioning performance coaches, administration, dietitians, coaches, faculty representatives, etc. A more detailed description of an athletic interprofessional care team will be provided later in the paper.

However, each of these individuals has their own specific knowledge that can be used for preventative care. For example, in a reactive care model, an injured athlete who is not adjusting to injury well would be referred to a licensed mental health professional after they begin to show signs of distress. In a stepwise preventative model, the athlete would have injury-preventative physical training, proper nutrition to prevent injury, been educated on coping strategies, and been given a list of possible resources before they become injured, bolstering their physical and mental resilience both before and after injury. Without a breadth of professional backgrounds engaged in athlete well-being from the beginning, athletic departments will likely only see increases in mental health crises and diminished well-being in their student-athletes.

## 4. Interprofessional Collaboration

The concept of collaborative practice has been advocated for across health and social care for over 50 years. However, there have been challenges to the wider acceptance and implementation of this model. Three landmark reports by the Institute of Medicine [22,23,24] identified issues with the healthcare system and made recommendations to design a safer health system and improve the quality of the care experience moving forward. One of these recommendations (or “rules”) were: “Cooperation among clinicians—Clinicians and institutions should actively collaborate and communicate to ensure an appropriate exchange of information and coordination of care” [23].

This “interprofessional” approach to care grew in importance globally; however, the healthcare workforce, who have traditionally been educated in professional “silos”, was challenged by the lack of skills and formal training needed for collaborative care [24]. To meet this need, the concept of Interprofessional Education (IPE) “Occurs when two or more professions learn about, from and with each other to enable effective collaboration and improve health outcomes” [25] (p. 130). To operationalize IPE in health professions programs, the Interprofessional Education Collaborative (IPEC) developed Core Competencies for Interprofessional Collaborative Practice in 2011, which were updated in 2016 and 2023 [26,27,28]. These core competencies (Table 1) have been widely recommended and adopted across the United States, including by 24 health professions accrediting bodies [29].

## 5. Interprofessional Care in Sport

Interprofessional care has been widely promoted in athletics [30,31,32,33,34,35,36]. The benefits of interprofessional care teams are emerging in the wider healthcare literature [37,38,39,40]. This is detailed by Ulrich and colleagues, who discuss how collaboration in sports medicine and sports science can positively impact medical patient outcomes such as better patient safety and satisfaction, improved healthcare, improved respect and trust between professions, decreased healthcare costs, higher workforce satisfaction, and lower staff turnover [36,41,42]. Though research in this area specific to NCAA athletic departments is still in the early stages, parallels can be drawn from the medical literature with regard to how interprofessional collaboration can benefit both student-athletes and staff. This may include a better focus on care for the whole person, better response to emergent situations, building trust and shared knowledge across professional domains, and improved well-being for student-athletes—the main goal for most, if not all, NCAA institutions. However, in order for these teams to function effectively, specific guidelines are needed to help navigate the complexity of interprofessional care.

## 6. Barriers to Implementing Interprofessional Care

Despite the push for an increase in interprofessional collaboration in university athletics, the fact remains that few universities have a formalized collaborative care team that follows the competency framework identified above. Several authors have surveyed sports medicine professionals about their attitudes and beliefs around interprofessional care, as well as barriers to implementing interprofessional teams in practice. Breitbach and colleagues reported that the teamwork principles of sport can inform care teams, stating “recognized positive aspects of teamwork in sport that can translate to improvement in care include clarity of purpose/goal, well-defined roles, communication and opportunities for practice and team development”, [43] (p. 10).

While attitudes were largely positive about interprofessional care in athletics, researchers identified multiple barriers to implementing IPC in sports medicine that largely stemmed from interpersonal and structural concerns. These included communication between professions, role ambiguity, differing values, ethical standards, traditional professional hierarchies, and commitment of stakeholders [31,32,34,43,44,45,46]. When discussing athlete well-being, where a care team widens to include various other professions outside of just sports medicine, it becomes evident that these barriers would likely only increase in complexity. Additional barriers such as navigating confidentiality, perceived power imbalances between professions, differing ethical codes, and scope of practice are likely to come up when navigating interprofessional care teams in a university athletic department [31,36]. Therefore, the following portion of this paper will take an applied approach, using a case vignette to describe how IPC was implemented at an NCAA Division 1 institution and how the interprofessional team utilized the IPEC core competencies to overcome the barriers described above.

## 7. Interprofessional Wellness Team (IWT)

### 7.1. Forming the Team

The following information will be provided from the perspective of the interprofessional team at an NCAA (National Collegiate Athletic Association) Division I university, from here on called the Interprofessional Wellness Team (IWT). The university is in an urban setting and is identified as a primarily white institute. This demographic is reflected in the general athletic staff and the members of the IWT. The athlete population, which consists of approximately 450 student-athletes from 18 sports, is made up of individuals from all backgrounds; however, it is predominantly white with a high number of international student-athletes. The demographic information provided here is crucial for contextualizing the clinical recommendations provided throughout the rest of this paper.

The Interprofessional Wellness Team (IWT) is made up of four core members, a licensed athletics mental health therapist, a sports dietitian, a doctoral candidate in clinical sports psychology, and an athletic trainer. All four core members are women. The IWT also consults with a broader group of individuals, including athletic administrators, academic coordinators, faculty in athletic training and psychology, sports performance, the director of the university counseling center, and sports medicine professionals. The specifics of how the IWT works with the larger group will be discussed in detail below. The IWT was conceptualized from ongoing informal conversations about supporting student-athlete wellness and was formalized following a request for well-being promoting programming for student-athletes from the athletic administration. The makeup of the IWT and larger consultation team is in line with recommendations from the interprofessional care team literature [3,36,43]. Of note, recent work in this area has emphasized the importance of including athletics administrators in interprofessional collaboration, as these individuals are crucial in developing policies and allocating resources [3]. The inclusion of athletic administrators in the IWT has allowed for increased access to resources that are used to support student-athlete well-being.

### 7.2. Identifying Barriers

The IWT identified four main barriers to working on an interprofessional care team and providing resources to student-athletes—issues of confidentiality, clarifying roles, communication, and sharing leadership responsibilities. Other barriers that were identified but the IWT felt were captured within the four main barriers included education on cultural humility and competency, education and training on interprofessional collaboration, and scheduling conflicts. Each of these barriers will be expanded upon in the context of the IPEC Core Competencies described below.

### 7.3. Teamwork in Action

The following case vignette will be used to contextualize how the IWT works in a NCAA sports context. A women’s basketball athlete is two months post-surgery for an anterior cruciate ligament (ACL) tear. Her athletic trainer (a core member of the IWT) has noticed the athlete has been consistently late to rehabilitation and has expressed concerns over the time of her return to play progression. When the athletic trainer asks the athlete how she is doing, the athlete states she is not sleeping very well and feels isolated from her team because she cannot practice or compete in games. The athletic trainer also notices the athlete seems to be losing weight fairly quickly and seems to have low energy and mood compared to preinjury attitudes. Additionally, her physical therapist has reported she has missed sessions, is not fully engaged in her rehabilitation exercises, is not completing exercises at home consistently, and is progressing slower than she should be. The athletic trainer concludes that the athlete is showing signs of injury maladjustment and may be experiencing feelings of depression. To help provide support for the athlete, the athletic trainer decides to bring in the other members of the IWT.

### 7.4. Values and Ethics

The first IPEC core competency is Values and Ethics [28]. One of the biggest barriers the IWT faces is the issue of confidentiality, particularly as it applies to navigating four different ethical codes. Each individual on our team must work within the ethical boundaries of their field. For example, the athletic trainer must abide by the National Athletic Trainer Association (NATA) Code of Ethics, the sports psychologist must follow the American Psychological Association (APA) Code of Ethics, the dietitian has the Academy of Nutrition and Dietetics (AND) Code of Ethics, and a licensed athletics mental health therapist may rely on the American Counselling Association (ACA) Code of Ethics. Each code may have different standards or best practices for maintaining confidentiality and personal information covered by HIPAA. Additionally, each governing body has a different, albeit overlapping, set of values that the professional is expected to uphold and abide by.

In the case presented above, the athletic trainer must decide the best practices for referring her athlete to the necessary resources. On our team, when the athletic trainer identifies the athlete as struggling with injury adjustment and displaying concerning symptoms of distress, they connect with either the sports psychologist or the athletics mental health therapist in two ways—we will differentiate between these two roles in the following section. First is by having a direct conversation with the athlete expressing concern for their well-being and asking if they are willing to speak with either the psychologist or therapist. If the athlete agrees, the athletic trainer will connect them with the provider via encrypted email. Additionally, the athletic trainer may coordinate with the provider to engage in a “warm hand-off” in the training room. The role of proximity in facilitating this, and other, processes will be discussed in a subsequent section.

In this case, the sports psychologist is introduced to the athlete via encrypted email and schedules the initial consultation. At this first session, the sports psychologist, bound by the APA Code of Ethics, discusses informed consent with the athlete, potential risks to privacy, and instances when they must break confidentiality, such as being a mandated Title IX reporter or if the athlete is in danger of harming themselves or others. These are all standard informed consent processes. However, to engage this athlete in multidisciplinary care and allow for care coordination, the sports psychologist includes a specific release of information (ROI) section on the consent form that allows the athlete to consent for them to speak to any number of individuals, including (but not limited to) the athletic mental health therapist, sports physician, athletic trainer, dietitian, coaches, physical therapy, or sports administrator. This ROI allows the clinician to ethically engage in a team-based approach to care. Another part of this conversation is letting the athlete know that the clinician is a member of the broader sports medicine team that meets weekly and therefore will be provided with broader updates on their progress from other individuals involved in their rehabilitation and can act as a touch point for the clinician to ask for more information or share updates with individuals they may otherwise not connect with.

If the athlete consents to the clinician speaking with other members of our team—in this case, the athletic trainer and the sports medicine team—the sports psychologist reaches out to the athletic trainer to schedule weekly meetings in a secure office or on telehealth platforms with the athletic trainer to discuss concerns and check in on progress. This is also a time in which they can coordinate care, such as the athletic trainer having the athlete complete an imagery or mindfulness exercise during rehabilitation. Additionally, because they consented to speaking with the larger sports medicine team, the sports psychologist can provide updates or coordinate care with other professionals such as physical therapy to support their work with the athlete. The sports psychologist ensures they share only necessary information about the progress of the athlete or concerns they have, thus ensuring that privacy is maintained.

If the athlete does not consent to the sports psychologist speaking with anyone on the IWT, things become more complicated; this is a key example of the importance of communication and IPC education. At the initiation of the IWT, each member shared the confines of their Code of Ethics and the limits to what they can share without proper documentation, such as an ROI. In this case, the athletic trainer has referred the athlete to the sports psychologist, therefore both parties know they are engaged in these services. However, because the athletic trainer is aware of the rules of confidentiality outlined in the APA Code of Ethics, they do not ask for information regarding progress or speak with the clinician, especially about that athlete. During the weekly sports medicine meeting, the sports psychologist can listen to updates about the athlete’s rehabilitation progression without breaking confidentiality and gain information that can aid case conceptualization or treatment planning.

As evidenced by the precarious nature of confidentiality on a team where each profession has a different code of ethics and values to adhere to, it takes continuous and honest conversations about ethical limits in order to protect the athlete while ensuring they are receiving the necessary resources to support their well-being. An interprofessional team should be aware of the unique nature of working in an athletics setting that may interfere with confidentiality. For example, the sports psychologist must navigate working with multiple individuals on the same team, working with coaches, conducting group or team sessions, or being in close proximity to colleagues in other professions. Therefore, setting clear boundaries between roles and discussions about confidentiality with athletes, coaches, and colleagues is a crucial aspect of the sports psychologist’s role. Additionally, the interprofessional team should also ensure that they work with each other to develop consent forms and ROIs that protect the athlete’s confidentiality and privacy while making it easy to coordinate care if the athlete consents to sharing information. This will likely involve communication with athletics administration and legal counsel to ensure that these documents fit within the legal and ethical scope of practice. And lastly, all members of the team must be educated on the limits of confidentiality as it pertains to the athlete’s safety and well-being. This is particularly important for athletes at risk for suicidality or who have been sexually assaulted. It is recommended that the care team develops a safety protocol for these at-risk athletes that clearly outlines reporting guidelines and avenues for connecting the athlete with crisis resources such as hotlines, a Title IX office contact person, or university counseling center emergency contact lines.

### 7.5. Roles and Responsibilities

In line with IPEC core competency #2, *Roles and Responsibilities* [28], one of the other major barriers the IWT faces is delineating between members of the team whose competencies overlap—such as the sports psychologist, athletic mental health therapist, and sports dietitian. In the case above, the athlete is presenting with a multitude of symptoms that could align with the roles of each member. For example, the athlete is socially isolated, losing weight quickly, and reporting low mood and fatigue. Each of these factors could be addressed by either the sports psychologist or the athletic mental health therapist. Additionally, the dietitian, sports psychologist, and athletic mental health therapist may all be competent in treating rapid weight loss in different ways, depending on if the athlete is purposefully restricting, not properly meal planning or consuming enough calories, or experiencing body image difficulties. To complicate things even more, the athletic trainer, sports psychologist, and athletic mental health therapist may all be suited to helping the athlete increase their rehabilitation adherence and increase motivation in recovery. In short, when working with athletes whose well-being is suffering, everyone may feel they are in the best position to help.

To overcome this barrier, the IWT has engaged in meaningful conversations regarding competencies and training. For the sports psychologist and athletic mental health therapist, this has been an ongoing discussion and will likely continue as the world of athletics continues to broaden from a more performance-focused model to a more well-being-focused model. One way we have approached this barrier is by ensuring every member can contribute to their role through their strengths. The sports psychologist has significant training and clinical experience working with injured athletes, while the athletic mental health therapist has more training and clinical experience in working with more severe mental health concerns in this age group through their other role in the University Counselling Center. An example of how we navigate these very similar roles using the case vignette would be to have the athlete first be referred to sports psychology due to the specific training and expertise in injury and rehabilitation psychology.

As described above, the sports psychologist will ask for consent to speak with other members of the sports medicine and IWT so that they can consult with others in their areas of strength. This may include checking in daily with the athletic trainer for progress updates and adherence, helping the dietitian identify potential disordered eating behaviors to consider in their work while addressing these behaviors using evidenced-based practices for disordered eating in athletes, and consulting with the athletic mental health therapist on adjustment and coping considerations. Moreover, if the athlete continues to regress and shows significant escalation in mental health symptoms, the sports psychologist is able to work with the athletic mental health therapist to provide higher acuity care, or even refer them to an inpatient or outpatient setting. The resources the athletic mental health therapist has through her position in the University counseling center afford her better access to tools and referrals than that of the sports psychology graduate student. The key to working on a team where individuals’ training and roles may overlap is to communicate clearly what each member’s strengths are and bolster your team member’s capabilities to use those strengths to benefit the well-being of the athletes.

A recent project the IWT has begun working on is the development of a referral protocol that clearly delineates the roles and responsibilities of each team member and provides specific instructions on when to refer the athlete to another team member who may be better suited for their strengths. A recommendation from these conversations is the development of a “wellness” intake form that an athlete who is struggling and would like services would complete. All four core members of the IWT would then review the intake form to assess who is most competent in the athlete’s main areas of concern. Though this discussion is ongoing, the goal remains to develop a system that best supports the athlete and utilizes the strengths of each member of the interprofessional team within the confines of their roles.

A central piece of addressing the barriers identified through IPEC core competency #2 [28] is the ability of each team member to reflect on potential gaps or areas of growth in their training in order to ensure the athletes are receiving the best care they can receive. Being aware of these gaps in training allows us to clearly identify our roles on an interprofessional team. An example of this that is being discussed frequently in sports is the difference in training between a clinical sports psychologist and a Certified Mental Performance Coach (CMPC), a certified performance specialist through the Association for Applied Sport Psychology. The educational paths, specific course work, and clinical supervision requirements of both credentials are very different but both are tasked with helping athletes succeed in their sport. The differentiation lies particularly in the scope of practice. Psychologists are licensed mental health professionals who can diagnose and treat a wide range of clinical problems and disorders. CMPCs do not have to be licensed—unless they are also a licensed mental health professional—and are certified to provide consultation to improve mental performance. CMPCs are unable to diagnose or intervene with clinical disorders and are instructed to refer athletes experiencing mental health concerns to a licensed professional. If both individuals are working on an interprofessional team together, as is becoming increasingly more common, clearly defining the roles of both positions to all members of the team is crucial to make certain the athlete is receiving the care they need. While this concept of acknowledging gaps in training may seem like it can create conflict on a team, taking a strengths-based approach to interprofessional collaboration, such as described above, will provide space for individuals with different training to consult and bolster your work, all the while centering the well-being of the athletes.

Another key part of IPEC core competency #2 is that of responsibility. Specifically, RR5 states “practice cultural humility in interprofessional teamwork [28] (p. 17)”. While conversations occur about role clarity, clinical training, and/or education, this specific responsibility should receive a significant amount of consideration. Cultural humility consists of three components—(1) a personal lifelong commitment to self-evaluation and self-critique of their own beliefs and cultural identities, (2) recognizing power imbalances and striving to address these imbalances in your work and in the community, and (3) institutional accountability [47]. Cultural humility adds to cultural competency, the process of learning about different cultural identities, ensuring that the individual is able to reflect on these differences in relation to their own experience. To truly provide excellent care and promote the well-being of all athletes, all members of the interprofessional care team must engage in cultural humility. It is the responsibility of the team to create space to reflect on their own identities, on the identities of athletes from various sociocultural backgrounds (e.g., sexual orientation, gender, race, ethnicity, or socioeconomic status), and how these identities intersect and interact with our own identities to impact the well-being of the athlete. Without a deeper understanding of how factors such as power imbalances between IWT professionals and athletes impact the services provided, care teams will not be able to provide holistic care to truly support athlete well-being.

### 7.6. Communication

The third IPEC core competency is *Communication* [28]; this competency may by far be the most important aspect of interprofessional collaboration. Without consistent, clear communication, then all other aspects of care discussed in this paper are likely to fall apart. Barriers that impact communication may be factors such as proximity of team members, a lack of community building in a department, and differing communication styles. The IWT has received significant resources to overcome the first two barriers—proximity and community building. In 2023, our athletic department opened an athlete wellness center with space for all individuals involved in promoting student-athlete well-being. This is, to our knowledge, the first center built specifically for athlete wellness, and includes office space for administration, sports nutrition, sports psychology, athletic counselling, sports medicine, and athletic academic advisors. In addition to office space, the center includes space for athletes to work on homework or attend study hall, technology classrooms for team sessions, a cafeteria that features a menu developed by the sports dietitian, and a “fueling station” for quick, healthy snacks before or after practice, recovery, or a game. The center was built as an addition to the main arena that also holds the athletic administration offices, most coaches’ offices, weight room, locker rooms, athletic training room, and multipurpose gym. In short, the athletes are able to access all of the resources they need in one building.

For our team, this solves the issues of proximity and community building, as each member is housed in one athlete-centered location. The close proximity allows us to communicate directly, face-to-face, rather than over email or cell phone. In the scenario discussed above, the athletic trainer has the ability to arrange warm hand-offs with sports psychology. The sports psychologist, whose office is adjacent to the athletic mental health therapist and sports dietitian, then has the ability to simply walk next door to collaborate with the other team members once consent is obtained. Additionally, with all offices in one place and open, direct communication lines, team members are able to adjust scheduling around each other and the athlete. The goal for scheduling is to bring athletes in quickly to needed resources once they are identified as at risk, and then, because athlete time is a major barrier to care, explore ways to reduce overall time commitment in hopes of increasing treatment adherence and reducing attrition. One way our team has approached the issue of time constraints is by combining aspects of care into one session. In this scenario, this may look like the sports psychologist helping the athlete engage in healing imagery practice during rehabilitation or scheduling a check-in during the last 15 min of treatment using a confidential space in the training room. Another way we have approached this issue in the context of injury rehabilitation is to have the sports psychologist provide skills training to the athletic trainers, where they have the opportunity to learn how to implement skills such as progressive muscle relaxation, imagery, self-talk, and mindfulness into their treatment plans. This aspect of interprofessional education not only reduces the time needed by the athlete, as they would not need as frequent sessions with a sports psychologist but also provides opportunities for athletes who may still be hesitant to engage in services such as sports psychology or athletic counselling to receive a higher standard of care.

The proximity afforded by our athlete center also has tremendously improved communication through community building. The athletic department consistently promotes opportunities for staff and coaches to engage with each other at events hosted in the space, such as all-staff meetings, committee meetings, and even diversity-equity-inclusion book clubs and staff baby showers. By using the space to create time for all individuals to come together, create relationships, and share expertise, it solidifies a community committed to promoting athlete well-being. The IWT uses this space for meetings to address athlete concerns and develop programming centered on athlete well-being. However, over and above the influence of more formal gatherings, it is the casual, daily interactions among staff, administration, coaches, and athletes that build relationships, trust, and collaboration.

While the importance of proximity is not to be underestimated in aiding communication among interprofessional teams, we acknowledge the privilege afforded to us by having this space dedicated to helping our athletes. Most universities, especially at the NCAA Division II and II levels, may not have the resources to allocate to such an undertaking. Therefore, athletic administrators should strive to create opportunities for connection throughout the year, especially for individuals who may remain siloed in a traditional athletic organization, such as counseling, sports medicine, and nutrition. Moreover, once an interprofessional care team is identified and established, standing meetings should be scheduled—our team meets weekly—to ensure that communication is carried out outside of just email or phone contact. When communicating through email, individuals should take the proper steps to protect the confidentiality of personal information. Athletic administrators should identify appropriate encryption steps and train all staff and coaches in this process.

Underlying all aspects of communication should also be the understanding that all individuals have different communication styles. Individual and cultural factors largely determine how we communicate with each other. These factors include aspects such as being from a non-Western culture, individuals where English is not their first language, gender differences, generational differences, accommodations for differently abled individuals (e.g., needing to use American Sign Language), and other unique aspects of language and communication such as using African American Vernacular English (AAVE). All ways of communicating are valid and deserving to be heard. Athletic departments should provide training and psychoeducation on different styles of communication and how to create community with each individual involved in helping athletes’ well-being, as well as improve communication with athletes from all backgrounds. This may involve collaborating with offices on campus such as diversity, equity, and inclusion or disability services to provide this information to the athletic department.

### 7.7. Teams and Teamwork

The final IPEC core competency is Teams and Teamwork [28]. When working in a team with multiple professionals, all with significant training and competency in their individual areas, it is not a surprise that navigating aspects of teamwork such as leadership can become complicated. Proper leadership and a common identified goal are crucial to the productivity of a team, and the same applies to athlete interprofessional care teams. In the case of the IWT, the common goal is simple—promote athlete well-being. This goal is reached in two avenues, the first being reactionary individual care for an athlete who is struggling, such as the athlete identified in the case vignette. Another approach we have taken is a preventive approach to promoting athletes’ well-being. Specifically, we have developed a wellness series for the student-athletes at our university. The first step in developing these series was to create a shared leadership model. In short, each member of the IWT identified an area that they specialized in, was consistent with stated organizational values, and promoted wellness. Examples included the sports psychologist leading a goal setting and imagery workshop, the dietitian leading a mindful eating workshop, the athletic trainer organizing for a women’s and men’s health physician to come speak to athletes, and the athletic mental health therapist hosting a stress-free night during finals week. In the shared leadership model, each member was tapped as the leader for their session(s), which included organizing and creating materials, promotion, and leading the session. The other team members provided additional support and identified ways to bolster the session with their own areas of expertise. Each session was held in the athlete wellness center, another benefit of having close proximity. So how would these wellness series sessions help an athlete such as the one identified in the case vignette? From a preventative perspective, if the athlete had attended these wellness series previously, it is likely that they would have a skill set developed before their injury that could be drawn upon to bolster their well-being during the injury process. Also, because all members of the IWT attended each session, the relationships and familiarity built at these times may increase the likelihood of the athlete reaching out for support without the athletic trainer’s intervention. Without a shared leadership model that allows each member to highlight their skills and competencies, the athlete may not have exposure to a full set of well-being-promoting experiences, putting them at greater risk of injury maladjustment and mental health concerns down the line.

Another key factor of teamwork is identifying a common goal and working collaboratively towards that goal using core values. This means creating a level of trust and understanding between all members of the team, which is carried out through collective buy-in of common values. With the overarching goal of promoting athlete well-being, our team leaned strongly on the organizational values of our Jesuit university and athletic department. The university value of Cura Personalis, or care for the whole person, is a firm guiding factor in the decisions we make as a team. Our athletic values of trust, care, and commitment to excellence also shape how we approach our work. By committing to upholding these values together as a team, the IWT provides a truly holistic approach to promoting athlete well-being.

Creating a well-functioning interprofessional team can take effort and time. One way to help guide the formation and improve the team’s effectiveness is by adopting a shared mental model. The Department of Defense (DoD) and the Agency for Healthcare Research and Quality (AHRQ) developed the Team Strategies and Tools to Enhance Performance and Patient Safety (TeamSTEPPS™) program, designed to integrate teamwork into practice using a shared mental model. TeamSTEPPS^TM^ defines a shared mental model as an “organizing knowledge structure of the relationships between the task the team is engaged in and how the team members will interact” [48] (p. 5). Teams that have a shared mental model anticipate and predict each other’s needs as well as, if needed, identify changes in the team, task, or teammates, and implicitly adjust strategies [48]. Having an organized structure may help the team navigate and adjust for other difficulties discussed thus far, such as role clarification and communication. Additionally, using this approach to leadership on interprofessional teams is essential and can occur in several ways: (1) formal leadership in coordinating teams-based roles defined in the organization or (2) informally as contingency teams informed by the specific context where any member of the team could serve in a leadership role [49]. As demonstrated above, the IWT followed the second approach, loosely described as a shared leadership model, where leadership is informed by context and all members were able to serve in a leadership role. In short, utilizing shared values in an organization can help identify the task and goals at hand—such as promoting athlete wellness—while a shared mental model can help with the organization and functioning of the interprofessional team, especially with regard to leadership, in order to reach that goal. Using these considerations as a starting point can help athletic departments feel confident and comfortable in developing their own interprofessional teams to improve athletes’ well-being.

## 8. Discussion

This paper describes how a wellness team at an NCAA Division I athletic department navigates interprofessional care to promote student-athlete well-being using the IPEC core competencies. A main barrier identified in the first competency, *Values* and *Ethics*, was the issue of confidentiality, which led to a discussion on how the IWT navigates differing ethic codes to share confidential and private information about athletes in the team. The next core competency, *Roles* and Responsibilities, was discussed through the barrier of identifying and clarifying roles in the IWT, especially among members with similar specialties such as an athlete mental health professional and a sports psychologist. Discussion around the third competency, *Communication*, was centered on how proximity to team members and community building is imperative to the function of the team. The last competency, *Teams and Teamwork*, expanded on how the use of a shared leadership model by the IWT allowed each member to utilize their unique training competencies to improve care for their athletes. Additionally, the importance of shared values and identifying a common goal through a shared mental model were highlighted. In short, a multitude of barriers to providing interprofessional care can be identified both in this paper and in the general interprofessional literature [31,32,34,42,44,45,46]. However, interprofessional teams in athletic departments who hope to improve the well-being of their athletes may find solutions to said barriers by integrating the IPEC core competencies.

This paper is centered around the experience of an IWT at their university; the barriers identified, solutions developed, and resources highlighted were discussed within the context of their specific university and athletic department. Recognizing that each institution and athletic department is unique, with unique resources, it is expected that they would all experience differing issues in developing and adopting an interprofessional care perspective. Therefore, the importance of context and individualizing the goals of the team to that context cannot be understated. The first step to this approach is to gain a deep understanding of the needs of the specific athletes at your university. Aspects of their sports experience that can influence well-being, such as access to mental health and sports medicine providers, provision and disbursement of scholarship money, and a culture of care in the athletic department, should all be explored within the context of not only the athletic department but also the university and surrounding community. Barriers to developing an interprofessional team and then additional barriers to providing care and promoting well-being by that team that are specific to those contexts should be identified and addressed. Moreover, those barriers must be overcome using the resources available within those contexts. The identification of contextual factors and specific barriers that influence the interprofessional team should be the first step for those invested in expanding care to their athletes through interprofessional collaboration.

Another common thread woven throughout this paper was the importance of cultural humility. Cultural and social factors such as race, gender, sexual orientation, socioeconomic status, and influence from coaches, teammates, and staff, all have varying impacts on the well-being of each athlete one works with. These factors, and many more, also impact every member of the interprofessional team. By committing to the practice of cultural humility, one can begin to develop an appreciation and understanding of their own identities, the identities of the athletes, and the identities of team members and how each of these interact to influence athlete well-being. This is simply one step to creating an inclusive culture of care within the interprofessional team and the athletic department at large. Unfortunately, microaggressions towards athletes of minorities are not uncommon on university campuses. Comeaux discusses how microaggressions identified by Division I athletes negatively influenced their self-concept, including making them question their own intellectual abilities and academic motivation [50]. Microaggressions have a multitude of additional negative impacts on well-being, such as lowered self-esteem, increased levels of stress, and increased risk of experiencing symptoms of anxiety, depression, and suicidality [51]. With the skyrocketing interest in protecting and promoting athlete well-being, it is evident that creating a safe, inclusive space for athletes to thrive in all aspects of their identity should be a high, if not the highest, priority for interprofessional teams in university athletics.

## 9. Recommendations

The primary purpose of this paper is to provide a practical framework and tangible recommendations for developing interprofessional teams as well as identifying and overcoming barriers in implementing collaborative care to improve athlete well-being. Table 2 summarizes the recommendations provided throughout this paper for each core competency.

In addition to the recommendations provided in Table 2, the members of the IWT also identified various other recommendations that can help facilitate interprofessional collaboration for athletes’ well-being. These include broader ideas and considerations for athletic administrators as well as interprofessional teams. The first suggestion for athletic administrators was to engage an organizational psychologist or other qualified professional to conduct a needs assessment for the athletic department, focused on what resources are missing that will facilitate athlete well-being. Once these needs are identified, athletic administrators can work with an interprofessional team to create programming or procedures to fill these gaps or hire individuals whose specialties are required. Administrators should consider obtaining input on how to improve care for their athletes from a multitude of stakeholders, such as coaches, staff, university administrators, and faculty. Most importantly, the athletes should be included in conversations around their care and the insight they provide should be seriously considered to show true investment in the well-being of the athletes. If the recommendations they provide are not implemented, open and honest communication with the athletes should provide clarification for these decisions in order to facilitate bidirectional trust. Next, athletic administrators should prioritize diversity, equity, and inclusion training in their departments for all staff, coaches, and athletes. Creating a culture of care as a part of an inclusive safe space takes an investment of both time and finances. For athletic departments that value the well-being of their athletes, the investment is well worth the pay-off. One last recommendation was for athletic administrators to review the NCAA Mental Health Best Practices [4], identify areas that may need bolstering, and collaborate with the interprofessional team to implement each best practice recommendation in their athletic department.

Another primary recommendation from the IWT for new interprofessional teams or athletic departments interested in developing their own wellness team is to engage in interprofessional education (IPE). IPE is defined as “when students (learners) from two or more professions learn about, from, and with each other to enable effective collaboration and improve health outcomes” [52]. However, historically, IPE has primarily occurred in academic units housing professional programs in medicine, nursing, pharmacy, and allied health professions [53]. However, many sports professionals were not trained in these programs and did not have the opportunity for formal IPE [36,42]. Therefore, the framework and recommendations provided in this paper serve as a starting point and efforts must be made to expand formal IPE to include sports professionals as well as provide interprofessional training for practicing professionals through continuing education.

In conclusion, while it may seem daunting at the offset, the practical recommendations provided in this paper provide a useful framework and call to action for individuals interested in developing an interprofessional wellness team in their university athletic department. Promoting athletes’ well-being is not a one-person job; it truly takes a team to provide holistic care to athletes.

## Figures and Tables

**Table 1:** IPEC Core Competencies for Interprofessional Collaborative Practice: Version 3 [28].

**Values and Ethics:** Work with team members to maintain a climate of shared values, ethical conduct, and mutual respect.
**Roles and Responsibilities:** Use the knowledge of one’s own role and team members’ expertise to address individual and population health outcomes.
**Communication:** Communicate in a responsive, responsible, respectful, and compassionate manner with team members.
**Teams and Teamwork:** Apply values and principles of the science of teamwork to adapt one’s own role in a variety of team settings.

**Table 2:** IPEC Core Competency [28] Recommendations for Interprofessional Teams to Improve Athlete Well-Being.

Competency	Recommendations to Consider
Values and Ethics	Identify and discuss limits to confidentiality at the initiation of an interprofessional team Disseminate and read each member’s ethical code Develop standard referral procedures for connecting athletes to different services Use encrypted email and telehealth platforms Include a release of information (ROI) in the consent forms of mental health providers Involve athletic administrators and legal counsel when developing new consent and ROI forms Develop standard language to use with athletes about the limits of confidentiality Develop safety protocol for at-risk athletes Maintain an up-to-date list of campus and community resources Develop procedures on how to connect athletes to external resources
Roles and Responsibilities	Discuss each member’s training and competencies at the initiation of the interprofessional team Identify individual and team areas of strength and weaknesses Discuss each team member’s potential gaps in training and whose training may fill these gaps Create ongoing space during meetings to reflect on individual and athlete identities and how they intersect and interact to impact athlete well-being Develop referral procedure that identifies each member’s role and responsibilities Develop specific instructions for referral when an athlete’s concerns become outside of the team member’s competencies Develop wellness intake form for athletes to initiate services and use an intake form to decide whose strengths best match presenting concern
Communication	Establish weekly meetings for the team to discuss general procedures for case collaboration Find space to meet face-to-face as a team on a regular basis Combine different interventions from team members (e.g., sports psychology and athletic training) into one session to save time for the athletes Address barriers to scheduling for athletes and problem solve solutions as a team Try to make space for daily, casual interactions among team and athletic department members Athletic administrators should identify appropriate encryption steps for communication and train all staff and coaches in this process Athletic departments should provide training and psychoeducation on different styles of communication Athletic departments should collaborate with on-campus offices (e.g., diversity, equity, and inclusion or disability services) to develop culturally sensitive communication practices
Teams and Teamwork	Identify common values of team members and develop a set of team values Use a shared mental model to develop team goals Invest time in developing preventative programming to improve athlete well-being, such as monthly wellness series or online psychoeducation modules Use a shared leadership model to highlight and utilize each team members specific strengths when developing programming

## Data Availability

Data are available upon request.
